# Spinal cord injury is associated with enhanced peripheral chemoreflex sensitivity

**DOI:** 10.14814/phy2.12948

**Published:** 2016-09-05

**Authors:** Amy T. Bascom, Abdulghani Sankari, M. Safwan Badr

**Affiliations:** ^1^ John D. Dingell VA Medical Center Detroit Michigan; ^2^ Department of Medicine Wayne State University Detroit Michigan; ^3^ Cardiovascular Research Institute Wayne State University Detroit Michigan

**Keywords:** Hypercapnia, hyperoxia, peripheral chemoreceptors, sleep‐disordered breathing, spinal cord injury

## Abstract

Sleep‐disordered breathing (SDB) is prevalent in individuals with chronic spinal cord injury (SCI), but the exact mechanism is unknown. The aim of this study was to investigate whether peripheral chemoreceptors activity is enhanced in individuals with chronic SCI compared to abled‐bodied control subjects using CO_2_ and O_2_ chemical tests. In protocol (1) 30 subjects (8 cervical [cSCI], 7 thoracic [tSCI] and 15 able‐bodied [AB]) were studied to determine the ventilatory response to hyperoxia during wakefulness in the supine position. In protocol (2) 24 subjects (6 cSCI, 6 tSCI, and 12 AB subjects) were studied to determine the ventilatory response to a single breath of CO_2_ (SBCO_2_). The chemoreflex response to SBCO_2_ was calculated as ∆V_E_/∆CO_2_ (L/min/mmHg). The ventilatory response to hyperoxia was defined as the % change in V_T_ following acute hyperoxia compared to preceding baseline. During hyperoxia SCI subjects had a significant decrease in V_T_ and V_E_ (63.4 ± 21.7% and 63.1 ± 23.0% baseline, respectively, *P* < 0.05) compared to AB (V_T_: 87.1 ± 14.3% and V_E_: 91.38 ± 15.1% baseline, respectively, *P* < 0.05). There was no significant difference between cSCI and tSCI in the V_T_ or V_E_ during hyperoxia (*P* = NS). There was no significant correlation between AHI and V_E_% baseline (*r* = −0.28) in SCI and AB (*n* = 30). SCI participants had a greater ventilatory response to an SBCO_2_ than AB (0.78 ± 0.42 L/min/mmHg vs. 0.26 ± 0.10 L/min/mmHg, respectively, *P* < 0.05). Peripheral ventilatory chemoresponsiveness is elevated in individuals with chronic SCI compared to able‐bodied individuals.

## Introduction

Spinal cord injury (SCI) is the second leading cause of paralysis and disability worldwide after stroke. It is estimated that traumatic SCI affects 54 cases per one million populations annually, which has not changed for several decades (Jain et al. 2015). While less than 10% of SCI patients will require mechanical ventilatory support beyond 1 year of injury patients with SCI are at increased risk for respiratory‐related complications due to impairment of neural outflow to critical respiratory muscles (Mansel and Norman 1990).

Sleep‐disordered breathing (SDB) is very common in patients with SCI and is related to injury level (Sankari et al. 2014d). In fact, SCI may be an independent risk factor for the development of SDB (Sankari et al. 2014c). The mechanisms underlying the increase in SDB in chronic SCI are not understood. One proposed mechanism is sleep‐related hypoventilation (Bauman et al. 2016). Individuals with cervical injury (cSCI), demonstrate significant sleep onset hypoventilation compared with patients with thoracic injuries (tSCI) and able‐bodied controls (Bascom et al. 2015), with subsequent frequent arousals, significant fluctuations in end‐tidal CO_2_ and O_2_ (P_ET_CO_2_, P_ET_O_2_, respectively), increased plant gain, and a narrowed CO_2_ reserve, all of which set the stage for breathing instability (Dempsey et al. 2004).

Altered chemoresponsiveness has been implicated in central SDB and breathing instability in able‐bodied subjects (Dempsey et al. 2004; Eckert et al. 2007; Salloum et al. 2010; Sankri‐Tarbichi et al. 2013) and individuals with SCI (Sankari et al. 2014c). Specifically, peripheral chemoreceptors have been proposed as a key component of the ventilatory feedback loop for producing breathing instability during sleep (Khoo et al. 1982, 2001; Longobardo et al. 1982). However, data on peripheral chemoreceptor responsiveness in SCI are lacking. The majority of studies have reported blunted hypercapnic ventilatory response (HCVR) (Kelling et al. 1985; Manning et al. 1992), which is a measure of peripheral and central chemosensitivity while other investigators have found no significant difference in sensitivity to steady‐state hypoxia and hypercapnia compared with able‐bodied controls (Ben‐Dov et al. 2009). Since peripheral chemoreceptors respond very rapidly, on a breath‐by‐breath basis, chemical stimuli that are rapidly administered and brief would best take advantage of their specific time response characteristics to avoid involving the slower responding central chemoreflexes (Dutton et al. 1967; Gelfand and Lambertsen 1973).

The hypotheses tested in this study are as follows: (1) subjects with chronic SCI will have a higher peripheral chemoreceptor contribution to eupneic breathing as evidenced by a greater magnitude of decrease in ventilation in response to transient hyperoxia; and (2) subjects with chronic SCI will have a heightened ventilatory response to transient hypercapnia compared to able‐bodied subjects. The combination of two interventions (brief hyperoxia and hypercapnia) would allow for the assessment of carotid body chemoreflex responses to O_2_ and CO_2_ stimuli. Results of this study have been previously reported in the form of abstracts (Sankari et al. 2014a).

## Materials and Methods

Protocols were approved by the Human Investigation Committee of the John D. Dingell Veterans Affairs Medical Center and Wayne State University (Detroit, MI) and written informed consent was obtained.

We studied adults (≥18 years old) with chronic SCI and able‐bodied participants if they met the inclusion and exclusion criteria. All subjects were instructed not to have alcohol, caffeine products or sedatives/narcotics on the day of the study.

*Inclusion Criteria:* Participants with SCI were included in the study if they were chronic (>6 months postinjury), and spanning the spectrum from cervical (cSCI, C4–C7) to thoracic levels (tSCI, T1–T6) (ASIA classification A–D). Able‐bodied subjects (AB) were recruited with similar demographics to the SCI group for age, body mass index (BMI) and gender.

*Exclusion criteria:* Participants were excluded from the study for any of the following: (1) pregnant or lactating females; (2) currently ventilator dependent or with tracheostomy tube in place; (3) history of cardiac disease including heart failure, peripheral vascular disease, or stroke; (4) history of head trauma resulting in loss of consciousness for more than 24 h; (5) advanced lung, liver, or chronic kidney disease; (6) extreme obesity, defined for this protocol as BMI > 38 kg/m^2^; or (7) other illness that would interfere with completion of the study.

Subjects first underwent overnight in‐lab polysomnography (PSG) to determine the presence or absence of sleep‐disordered breathing (apnea hypopnea index, events/hour; AHI). PSG studies were scored according to American Academy of Sleep Medicine (AASM) 2012 recommended criteria (Berry et al. 2012).

On a separate visit, subjects arrived at the lab between 10 am and 4 pm for study. Studies were performed in the supine position during wakefulness. Instrumentation included electrocardiogram (ECG), electroencephalogram (EEG), electrooculograms (EOG) and chin electromyograms (EMG) using the International 10–20 system of electrode placement (EEG: C3‐A2 and C4‐A2; EOG: O‐A2). Subjects wore a nasal mask connected to a pneumotachometer (Hans Rudolph, Model 3700A, Shawnee, KS) that measured airflow. Tidal Volume (V_T_) was determined via integration of the pneumotachometer flow signal. End‐tidal carbon dioxide (P_ET_CO_2_), end‐tidal oxygen (P_ET_O_2_) levels, and inspired O_2_ levels (FiO_2_) were measured with CO_2_ and O_2_ gas analyzers (Vacumed Model 17515 and 17518, respectively, Ventura, CA). Pulse oximetry was measured by an ear probe (Biox 3740, Datex‐Ohmeda Inc, Madison, WI). Respiratory effort was measured by respiratory inductance plethesmography (RIP) belts placed on the chest and abdomen (Q‐RIP, Braebon Medical Corp., Ogdensburg, NY). Ventilation data from the pneumotachometer, pulse oximeter, and gas analyzers were digitized and analyzed using a PowerLab Data Acquisition System (Model 16SP, ADInstruments Inc., Colorado Springs, CO). Electroencephalograph, EMG, EOG, ECG, and respiratory effort were recorded and analyzed via the Comet PSG system (AS40 amplifier) or Heritage II system (Grass Technologies, Warwick, RI). EEG was used to verify that subjects remained in stable wakefulness during interventions. The medical adhesive tape was placed over subject's lips to help prevent mouth breathing. Spontaneous ventilation was recorded for a minimum of 15 min before any intervention. Subjects were instructed that CO_2_ or O_2_ would intermittently be administered through the nasal mask for a short period, in random order, and that they were to breathe through their nose. If mouth breathing occurred, the tape was replaced, and the subject was again instructed not to breathe through their mouth. The order of interventions (hyperoxia vs. single breath CO_2_) was randomized to eliminate order effect.

### Hyperoxia methods

**Figure 1:**
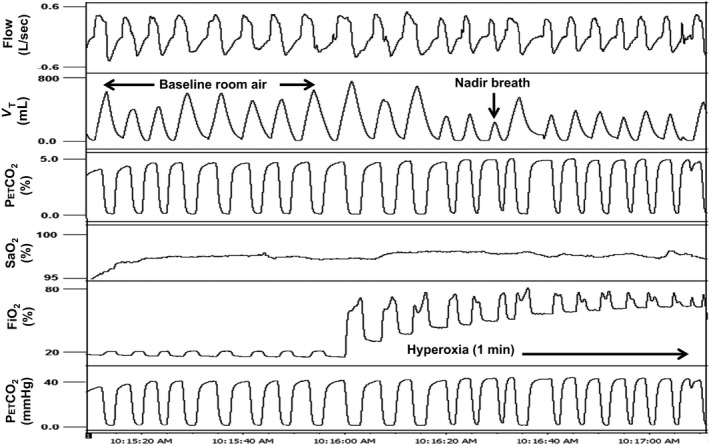
A representative polygraph of approximately 1 min hyperoxia trial in a 38 year old male cervical SCI subject (BMI 28.2 kg/m^2^). Baseline ventilation on room air preceding hyperoxia is followed by a striking decrease in tidal volume during hyperoxia administration. V_T_, tidal volume; P_ET_CO_2_, end‐tidal CO_2_; P_ET_O_2_, end‐tidal O_2_; SaO_2_, oxygen saturation; FiO_2_, concentration of inspired O_2_.

**Figure 2:**
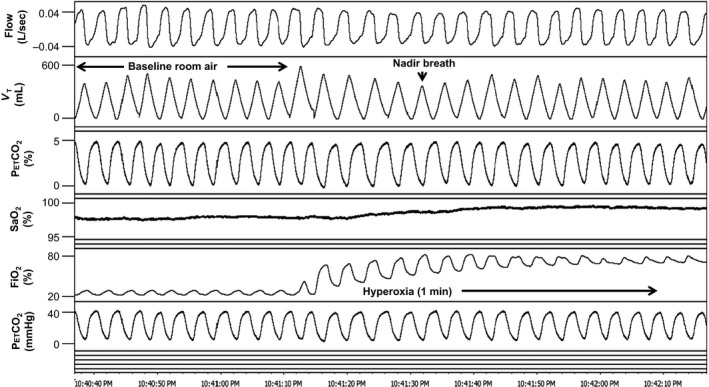
A representative polygraph of approximately 1 min hyperoxia trial in a 56 year old male able‐bodied subject (BMI 25.8 kg/m^2^). Baseline ventilation on room air preceding hyperoxia is followed by a smaller decrease in tidal volume during hyperoxia administration compared to that in Figure 1. V_T_, tidal volume; P_ET_CO_2_, end‐tidal CO_2_; P_ET_O_2_, end‐tidal O_2_; SaO_2_, oxygen saturation; FiO_2_, concentration of inspired O_2_.

Oxygen was bled into a port on the mask through O_2_ tubing attached to a gas tank containing 100% O_2_. Flow was quickly increased to 12–15 L/min until inspired O_2_ values reached ≥50% in the mask. O_2_ administration was continued for approximately 1 min followed by 5 min of room air breathing between trials. Trials were repeated three times. A representative example of a hyperoxia test in a cSCI subject is depicted in Figure 1 and an AB subject in Figure 2.

Analysis consisted of comparing the average of 10 baseline room air breaths immediately preceding hyperoxia with the VT nadir breath within the first 30 sec of hyperoxia. Results of three trials per subject were averaged. In one instance in a cSCI subject, only two reproducible trials were obtained, so, in this case, the average of two trials was used. Ventilation (minute ventilation [V_E_], V_T_, and frequency) for the nadir breath during hyperoxia was expressed as a percentage of the baseline room air ventilation. Nadir hyperoxia ventilation was compared between SCI and AB subjects. Also, average time (in seconds) to nadir breath during hyperoxia and average inspired % O_2_ (FiO_2_) were calculated and compared between groups.

A subanalysis was performed to compare the response to transient hyperoxia in cervical versus thoracic SCI subjects to determine if spinal injury level influences the response. Also, analyses were completed for all subjects to determine if AHI correlates with the ventilatory response to hyperoxia.

### Single breath CO_2_ methods

**Figure 3:**
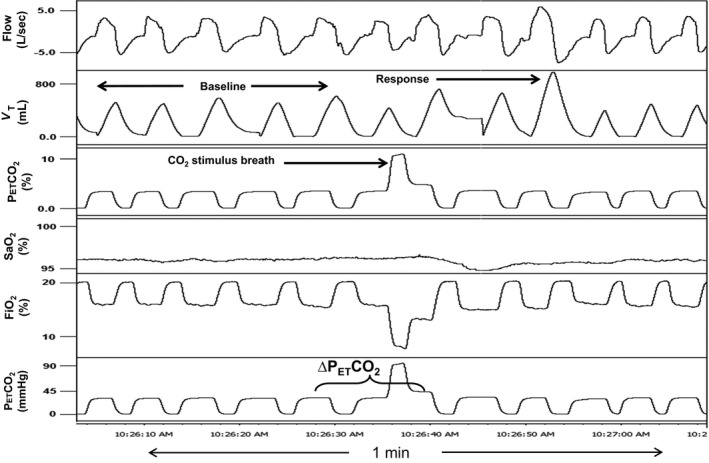
A representative polygraph of a single breath CO_2_ test in a 28 year old male cervical SCI subject (BMI: 31.5 kg/m^2^). Baseline room air ventilation is compared with the response breath (largest V_T_ within five breaths of CO_2_) after administration of 1 breath of CO_2_ (“CO_2_ stimulus breath”). Note the large increase in tidal volume in the response breath after CO_2_ administration in an SCI subject compared to the response breath in Figures 1 and 2, an able‐bodied subject. V_T_, tidal volume; P_ET_CO_2_, end‐tidal CO_2_; P_ET_O_2_, end‐tidal O_2_; SaO_2_, oxygen saturation; FiO_2_, concentration of inspired O_2_. ∆P_ET_CO_2_: P_ET_CO_2_ after CO_2_ administration minus the average P_ET_CO_2_ for baseline room air breaths before test.

**Figure 4:**
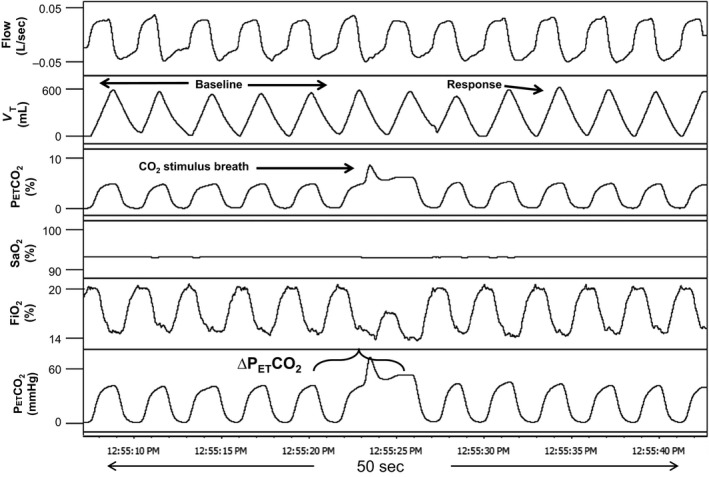
A representative polygraph of a single breath CO_2_ test in a 30 year old male able‐bodied subject (BMI: 22.9 kg/m^2^). Baseline room air ventilation is compared with the response breath after administration of 1 breath of CO_2_ (“CO_2_ stimulus breath”). Note the relatively small increase in tidal volume in the response breath (largest V_T_ within five breaths of CO_2_) after CO_2_ administration of the able‐bodied subject in this figure, compared to the response in Figure 1, an SCI subject. V_T_, tidal volume; P_ET_CO_2_, end‐tidal CO_2_; P_ET_O_2_, end‐tidal O_2_; SaO_2_, oxygen saturation; FiO_2_, concentration of inspired O_2_. ∆P_ET_CO_2_: P_ET_CO_2_ after CO_2_ administration minus the average P_ET_CO_2_ for baseline room air breaths before test.

Single breath CO_2_ tests (SBCO_2_) using the instrumentation and conditions described above were performed. While breathing room air, CO_2_ was bled into a port on the mask via small bore tubing connected to a gas blender (Model PMR4, Orangeburg, NY), which in turn was connected to a gas tank containing 40% CO_2_ balanced with N_2_. Gas flow was adjusted to administer approximately 8–10% inspired CO_2_ during expiration of one breath to load the circuit and continued flowing until peak inspiration of the following breath when it was abruptly terminated. Figures 3 and 4 demonstrate trials of SBCO_2_ in a cSCI and AB subject, respectively. Trials were preformed three times with 2–3 min between trials.

Analysis consisted of averaging the five breaths of baseline room air ventilation (V_T_, V_E,_ and P_ET_CO_2_) immediately before CO_2_ administration for comparison with the “response breath”, which was taken as the largest breath based on V_T_ within the first five breaths after administration. The chemoreflex response to a single breath of CO_2_ was calculated as ∆V_E_/∆CO_2_ (L/min/mmHg). Also, the V_T_ and V_E_ of the response breath were expressed as a percentage of the average V_T_ and V_E_, respectively, for baseline room air breaths. Time from CO_2_ breath to response breath was calculated in seconds. Results were averaged for three trials in each subject and outcomes were compared between SCI and AB groups.

A subanalysis was performed to compare the response to a single breath of CO_2_ in cervical versus thoracic SCI subjects to determine if spinal injury level influences the response. Also, the analysis was performed for all subjects to determine if AHI correlates with SBCO_2_ response.

### Statistical analysis

*T*‐tests were used to compare outcome measures and demographic data between the SCI and AB groups or between cSCI and tSCI when data were normally distributed. If data were not normally distributed, nonparametric tests were employed (SigmaPlot 12.1). Pearson Product Moment Correlation was performed to determine the relationship between AHI and ventilatory response to chemical stimuli. All data are reported as mean ± SD and significance was set at *P* < 0.05.

## Results

### Hyperoxia in SCI versus able‐bodied subjects

**Figure 5:**
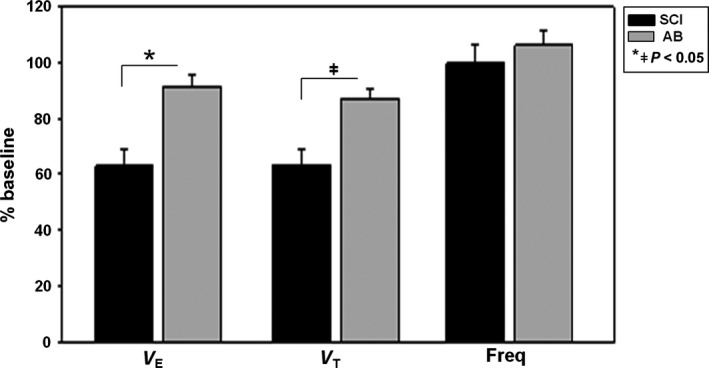
Minute ventilation (V_E_), tidal volume (V_T_) and frequency (Freq) during hyperoxia administration in spinal cord injury (SCI) (*n* = 15) and able‐bodied (AB) subjects (*n* = 15) are expressed as a percentage of baseline room air ventilation before intervention. SCI subjects had a significant decrease in V_E_ and V_T_ with hyperoxia (*P < 0.001, ^ǂ^ *P* = 0.001) compared to AB subjects. There was no significant change in frequency in either group (*P* = 0.43) using *t*‐test. Values are mean ± SEM.

Fifteen subjects with chronic SCI and 15 AB subjects with similar demographics (Table 1) were studied to determine the ventilatory response to hyperoxia. Figure 5 illustrates that SCI subjects had a significant decrease in V_T_ (63.4 ± 21.7% baseline) and V_E_ (63.1 ± 23.0% baseline) with hyperoxia compared to AB subjects (V_T_: 87.1 ± 14.3% baseline, V_E_: 91.38 ± 15.1% baseline) while frequency was not different in either group. The time from initiation of hyperoxia to the nadir breath was similar in both groups (SCI: 20.2 ± 3.6 sec. AB: 18.3 ± 5.4 sec, *P* = 0.26) as was the average FiO_2_ administered for all hyperoxia trials (SCI: 73.0 ± 11.5% vs. AB: 71.2 ± 8.7%, *P* = 0.63). There was no significant correlation between AHI and V_E_% baseline (*r* = −0.28) in SCI and AB (*n* = 30).

**Table 1:** Subject characteristics

	Hyperoxia		Single breath CO_2_	
	SCI	Able‐bodied	SCI	Able‐bodied
*N*	15	15	12	12
Age (years)	40.7 ± 13.4	41.3 ± 18.3	39.8 ± 13.2	41.3 ± 16.6
BMI (kg/m^2^)	25.8 ± 5.4	27.4 ± 4.1	27.0 ± 5.1	26.9 ± 4.1
Gender (M/F)	11/4	10/5	9/3	9/3
AHI (events*/*hour)	20.0 ± 17.4	11.7 ± 18.2	21.2 ± 19.3	12.7 ± 20.3
Injury level (cervical/thoracic)	8/7	–	6/6	–

All Mean data* ±* sd. BMI, body mass index; AHI, apnea hypopnea index. No significant difference in age, BMI, gender, or AHI (*P* > 0.05) using *t*‐test.

### Hyperoxia in cervical versus thoracic SCI subjects

To determine the effect of spinal injury level on ventilatory response to transient hyperoxia, cSCI (*n* = 8) versus tSCI (*n* = 7) subjects were compared (Table 2). There was no significant difference in the nadir breath V_T_ or V_E_ or frequency expressed as a % of baseline ventilation during hyperoxia trials. Results are detailed in Table 3. There was no difference in time to response to hyperoxia (cSCI: 21.4 ± 3.8 sec, tSCI: 18.7 ± 30 sec; *P* = 0.8), or average trial FiO_2_ (cSCI: 72.9 ± 9.2%, tSCI: 73.2 ± 14.4%; *P* = 0.96). There was no significant correlation between AHI and hyperoxia response in SCI subjects (*n* = 15) (*r* = −0.40).

**Table 2:** Cervical and thoracic SCI subject characteristics

	Hyperoxia		Single breath CO_2_	
	Cervical	Thoracic	Cervical	Thoracic
*N*	8	7	6	6
Age (years)	42.5 ± 13.0	38.7 ± 14.7	40.5 ± 11.0	39.0 ± 16.1
BMI (kg/m^2^)	24.4 ± 5.7	27.4 ± 4.9	26.2 ± 5.4	27.8 ± 5.2
Gender (M/F)	7/1	4/3	5/1	4/2
AHI (events/hour)	29.0 ± 16.5	9.6 ± 12.4*	33.2 ± 17.0	9.2 ± 13.6ǂ

All Mean data* ±* sd. BMI, body mass index; AHI, apnea hypopnea index. No significant difference in age, or gender. (*P* > 0.05). AHI is significantly higher in cervical versus. thoracic SCI subjects (*P = 0.02, ^ǂ^ *P* = 0.03) using *t*‐test.

**Table 3:** Cervical versus thoracic SCI chemoresponse ventilation

	Hyperoxia		*P* value	Single breath CO_2_		*P* value
	Cervical	Thoracic		Cervical	Thoracic	
*N*	8	7		6	6	
V_E_ (% baseline)	54.5 ± 21.3	72.9 ± 22.1	0.13	172.9 ± 40.3	153.3 ± 23.2	0.33
V_T_ (% baseline)	55.8 ± 20.1	72.1 ± 21.5	0.15	208.9 ± 52.9	157.6 ± 30.4	0.07
Frequency (% baseline)	99.9 ± 33.6	99.1 ± 13.0	1.0	–	–	
SBCO_2_ (L/mmHg)	–	–		0.82 ± 0.48	0.74 ± 0.39	0.75

All data Mean* *± sd. sbco_2_, Ventilatory response to a single breath of CO_2_ (∆V_E_/∆CO_2_). No significant difference in any chemoreflex responses between cervical and thoracic SCI subjects.

### Single breath CO2 in SCI versus able‐bodied subjects

**Figure 6:**
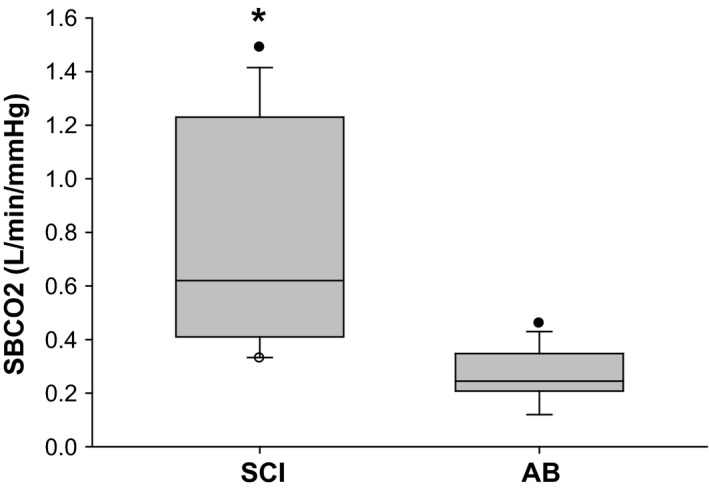
The ventilatory response to a single breath of CO_2._ Note that SBCO_2_ was significantly higher in SCI subjects (*n* = 12) compared to able‐bodied (AB) subjects (*n* = 12) (*P < 0.001) using Mann–Whitney Rank Sum Test. The horizontal line in the boxes represents the median, and the bottom and top of the boxes the 25th and 75th percentiles, respectively. I bars represent the upper adjacent value (75th percentile plus 1.5 times the interquartile range) and the lower adjacent value (corresponding formula below the 25th percentile), and the dots represent the outliers.

Twelve subjects with chronic SCI and 12 AB subjects with similar demographics were studied to determine the response to a single breath of hypercapnia. The ventilatory response to SBCO_2_ (∆V_E_/∆CO_2_) was significantly higher in the SCI group compared with the AB group, as detailed in Figure 6. Tidal volume for the response breath after CO_2_ administration, expressed as a percentage of baseline room air ventilation, was also significantly increased in SCI compared to AB subjects (183.2 ± 49.1% vs. 125.7 ± 13.6%, respectively, *P* < 0.05). Similarly, V_E_ increased to a greater degree in SCI subjects compared to AB subjects (163.1 ± 33.0% vs. 118.5 ± 4.8%, respectively, *P* < 0.05). The average inspired CO_2_ for SBCO_2_ trials was not different between SCI and AB groups (8.7 ± 1.6% vs. 8.0 ± 1.3%, respectively, *P* = 0.24) nor was the time from CO_2_ administration to the response breath (SCI: 10.6 ± 4.2 sec; AB: 11.9 ± 4.1 sec, *P* = 0.44). There were no clinically relevant changes in oxygen saturation during CO_2_ administration compared with baseline room air breathing in either group (SCI: SaO_2_ at baseline 96.0 ± 1.0%, SaO_2_ during administration of CO_2_ up to response breath 95.5 ± 1.3%, *P* = 0.02; AB: SaO_2_ at baseline 97.2 ± 1.0%, SaO_2_ during administration of CO_2_ up to response breath 96.8 ± 0.9%, *P* = 0.1). No significant correlation was found between AHI and SBCO_2_ response in SCI and AB subjects (*n* = 24) (*r* = 0.27).

### Single breath CO_2_ in cervical versus thoracic SCI subjects

To determine the contribution of spinal injury level to the response to a single breath of CO_2_, cervical (*n* = 6) versus thoracic (*n* = 6) SCI subject's responses were compared. Table 2 describes the characteristics of the cervical and thoracic SCI groups. There was no difference in SBCO_2_ (∆V_E_/∆CO_2_) response between cSCI and tSCI subjects (Table 3). Similarly, no difference was found in V_E_ or V_T_ expressed as a percentage of baseline ventilation between injury levels (Table 3). In addition, no difference was found in time from the inspiration of CO_2_ to response between cSCI (12.0 ± 5.6 sec) and tSCI subjects (9.2 ± 1.8 sec; *P* = 0.28). The inspired CO_2_ level for trials was not different between groups (cSCI: 8.9 ± 1.4%, tSCI: 9.2 ± 1.8; *P* = 0.59). There was no significant correlation between AHI and SBCO_2_ response in SCI subjects (*n* = 12) (*r* = 0.27).

## Discussion

The purpose of this study was to determine the peripheral chemoreflex responses to brief hyperoxia and hypercapnia in chronic SCI and AB subjects. The main findings of the study were: (1) subjects with SCI have a greater magnitude of reduction in ventilation in response to brief hyperoxia than AB; (2) sCI participants had a greater ventilatory response to a single breath of CO_2_ than AB; (3) there was no difference in peripheral chemoreceptor response to brief hyperoxia or hypercapnia in cervical versus thoracic SCI; (4) peripheral chemoresponsiveness was not significantly correlated with AHI in SCI and AB subjects.

### Peripheral chemoreceptor response to transient hyperoxia

In this study, we utilized brief hyperoxia to suppress the tonic drive of the carotid body to assess the putative contribution the carotid body to eupneic ventilation in SCI and AB subjects. Peripheral chemoreceptors located in the carotid bodies send a tonic excitatory input to central respiratory centers and contributes to eupneic ventilatory drive during sleep when wakefulness inputs from higher brain centers are absent (Forster et al. 2000). The receptors have been postulated to be involved in the development of SDB (Khoo et al. 1982; Dempsey et al. 2004). We noted that AB subjects had a decline in V_E_ of ~9% with, in agreement with historical data. Published studies report that hyperoxia is associated with reduced V_E_ by ~10%, which is mediated by the peripheral chemoreceptors (Dejours 1962). In contrast, hyperoxic ventilatory decline in SCI subjects was ~37%. This finding indicates a higher contribution of the carotid bodies to eupneic ventilation in individuals with chronic SCI relative to able‐bodied individuals.

### Peripheral chemoreceptor response to brief hypercapnia

We noted higher ventilatory response to SBCO_2_ in the SCI group compared with the AB group, indicative of higher peripheral chemoresponsiveness in patients with chronic SCI. Studies of chemosensitivity to CO_2_ in an animal model, utilizing an isolated perfusion of the carotid body, found that central chemoreceptors respond in a slower fashion to CO_2_ stimulation compared to the carotid body (30.9 sec vs. 19.6 sec, respectively) (Blain et al. 2010). Therefore, the carotid body contributes significantly to the ventilatory response to brief oscillations in CO_2_ such as were administered in this study. The methods used to determine the ventilatory effect of transient hypercapnia in this study utilized the response characteristic unique to peripheral chemoreceptors. McClean et al. using similar methods to determine the response to transient CO_2_ in able‐bodied adults (26 males and 26 females) found the average response to be ~0.34 L/min/mmHg (no significant gender or age effect was found), whereas able‐bodied subjects in this study had an average response of ~0.26 L/min/mmHg (vs. SCI with ~0.78 L/min/mmHg) (McClean et al. 1988). One reason for the slightly higher response in the previous study could be explained by the use of higher inspired CO_2_ (13%) while this study utilized 8‐9% CO_2_. However, the time response in both studies was consistent, with an average of 10–12 sec from CO_2_ administration to response.

#### Methodological considerations

Several considerations may influence the interpretation of the findings in this study. First, ventilatory changes in response to brief hyperoxia and hypercapnia tests could not be due to the sleep state or position, given the tests were done during wakefulness in the same position. Second, differences in the magnitude of ventilatory response could not be explained by differences in gender distribution or the different prevalence of SDB among the two groups. The study included a similar number of men and women in both SCI and AB groups. Although animal studies have shown gender specific carotid bodies ventilatory responses, studies in humans have demonstrated no gender difference in ventilatory response to hypoxia (Tarbichi et al. 2003; Genest et al. 2004). Ventilatory responses following hyperoxia and hypercapnia could be influenced by the severity of SDB. However, when we compared a subset of subjects from SCI group, the cervical SCI subjects had similar ventilatory responses to hyperoxia and single breath CO_2_ despite more severe SDB and significantly higher AHI in the cervical group. Therefore, robust chemosensitivity in the SCI group was due to the spine injury per se and not to the underlying SDB. Third, there was significant variability in the ventilatory responses to chemical tests (O_2_ and CO_2_), which may have attenuated the differences between cervical and thoracic SCI, particularly during sleep. We have shown recently that cervical SCI group decreased minute ventilation significantly following hyperoxia in comparison to thoracic SCI and AB groups (Sankari et al. 2014a, 2015). Therefore, there may be real difference between these two groups of SCI but this study was insufficiently powered to detect it. Moreover, the single breath CO_2_ stimulation is mainly hypercapnic test, as we did not add oxygen to prevent any transient hypoxia. However, the test was only for one breath and the method used in the study is an established method for the use of single breath CO_2_ test using McClean et al. (1988) study (McClean et al. 1988). To address this possibility, it need be tested in future using CO_2_ gas under hyperoxia. Finally, while not all subjects had both experiments, there was an overlap between the two experiments (all 12 AB subjects who had SBCO2 had hyperoxia experiments, while 10 of 12 SCI had both experiments).

#### Putative mechanisms and implications

Increased peripheral chemoreceptor activity and responsiveness in patients with SCI could not be explained by age, gender, or the presence of SDB. Thus, it is likely due to the injury per se or a long ‐term consequence of SCI. One possible explanation is that patients with SCI are likely to suffer from repetitive episodes of hypoxia over several years, due to SDB, impaired cough, retention of secretions and frequent pulmonary infections (Sankari et al. 2015). Animal studies suggest that chronic intermittent hypoxia (CIH), results in the development of sensory long‐term facilitation (LTF), which manifests as increased peripheral chemoreceptor activity and enhanced propensity to develop LTF following acute intermittent hypoxia (Peng et al. 2003). Our recent finding of augmented ventilatory LTF in patients living with SCI corroborates this explanation. However, the aforementioned interpretation is a speculation awaiting empiric proof.

In conclusion, individuals with chronic SCI have an increased peripheral chemoreceptor gain and a heightened reliance on the carotid body for maintenance of eupneic ventilation independent of the level of injury.

## Conflict of Interest

None declared.
